# An approach designed to fail deaf children and their parents and how to change it

**DOI:** 10.1186/s12954-024-01039-1

**Published:** 2024-07-10

**Authors:** Tom Humphries, Gaurav Mathur, Donna Jo Napoli, Christian Rathmann

**Affiliations:** 1 Department of Communication, University of California at San Diego, La Jolla, CA 92093 USA; 2 Department of Linguistics, Gallaudet University, Washington, DC 20002 USA; 3 Department of Linguistics, Swarthmore College, Swarthmore, PA 19081 USA; 4 Department of Deaf Studies and Sign Language Interpreting, Humboldt- Universität zu Berlin, Berlin, Germany

**Keywords:** Deaf children, Sign languages, Cognitive health, Linguistic deprivation

## Abstract

The matter of raising and educating deaf children has been caught up in percepts of development that are persistently inaccurate and at odds with scientific research. These percepts have negatively impacted the health and quality of life of deaf children and deaf people in general. The all too prevalent advice is to raise the child strictly orally and wait to see what happens. Only when the child is seriously behind is a completely accessible language – a sign language – introduced, and that is far too late for protecting cognitive health. The medical profession, along with others, needs to offer parents better advice and better supports so that neither the children nor their parents wait and watch as the oral-only method fails. All must take responsible action to assure an approach that succeeds.

## Background: updating what we know

A little over a decade ago in this journal, Humphries and colleagues [1] outlined the harms done to deaf children (“deaf children” is used inclusively here to cover children with a wide range of hearing loss, including those who are hard of hearing) by the practice of raising them orally with zero tolerance to the use of other evidence-based approaches – specifically, the introduction of sign language as soon as deafness is detected. We catalogued medical acts that have harmed the individual and society, including but not limited to:

- failure to inform (we made and justified the claim that parents are often not fully informed concerning language development in deaf children at the time when they are asked to make decisions that affect their children’s cognitive health, offering examples in a 2011 book [2], thus violating the legal principle known as the informed consent doctrine),
- misinforming and coercing behavior counter to the child’s welfare (we argued that incorrect information and coercive advice are often given to parents [3], among many),
- abnegation of trust (we argued that parents who follow misinformed medical advice and find their children falling behind hearing peers, experience a delay in facing their children’s needs [4], and often lose their faith in the very professionals who are supposed to be guiding them),
- as well as exposing the child to the many risks of overdependency on cochlear implant (CI) surgery (see [5–8], among many).

We placed the responsibility for these harms with medical and health-science professionals.

In the present article we give a brief history of how the zero-tolerance approach came about and why it should not be allowed to continue. Following that, we bring the reader up to date on relevant issues since the 2012 article. In one section we offer an update of the harms of a narrow deficit-centered approach. In another we give an update of the risks of surgery. Then we outline some of the major benefits of signing to deaf children, with or without a CI. Reinforcing the 2012 article, the present work ends with an overview of what changes need to be implemented at this stage.

## A history of harms: an approach designed to fail

Throughout history, in almost every part of the globe, models of deaf children’s needs regarding language and cognitive development have focused on hearing loss and the necessity of speech; from the moment hearing loss is identified, a quest ensues to find ways to help deaf children overcome inherent disadvantages accessing and processing spoken language [9]. In the modern world, replacing hearing, replacing the auditory pathway to language, too often becomes the sole goal [10]. Deaf children are faced with trying to develop spoken language with critically inaccessible input or degraded exposure to such input [11]. This approach has many limitations; listening technologies leave wide and unpredictable variation in their effectiveness, a child’s acoustic channel is subject to varying degrees of functionality, and relying on the child’s access to linguistic input via speechreading (lipreading) is highly risky [12].

The mentality that understands acquisition and development of language for deaf children solely as a search for restorative practices stems from a deeply held belief that the state of being without hearing and speech is catastrophic [13–15], a belief that persists today [16]. That belief is responsible for a long and persistent history of mistreatment of deaf children from birth to adulthood; in the end, only a small minority of deaf children acquire spoken language naturally with or without cochlear implants (CIs), as outlined in a study in 2013 [17]. Indications that the results of the 2013 study still hold are a decade of studies since that try to establish ever-increasing improvement in CIs, and the lack of studies during this decade that provide evidence that spoken language acquisition by deaf children with CIs proceeds as well and as naturally as acquisition among typically hearing peers.

Even in countries with a better understanding that sign languages are natural human languages and that delayed acquisition of language has detrimental effects on the architecture of the brain, most deaf children are raised in an oral-only environment, often with a CI (or bilateral implants) [18]. Many experience inadequate exposure to language, whether a spoken language or a sign language [19]. Parents, medical professionals, hearing and speech specialists, and others usually insist that children – given time, technology, and certain hearing and speech interventions – will develop necessary language and cognitive abilities [20]. It is only when the child demonstrates significant struggle in achieving developmental milestones that sign language is introduced (if ever), and remediation is attempted [21]. Remediation even for hearing children that have been left behind is rarely successful; instead, better educational practice is required to eliminate the need for it [22, 23]. The same should hold true for deaf children.

Even in countries where medical professionals and hearing-and-speech specialists understand that there is great cognitive risk to the deaf child who does not have sufficient input and exposure to language, these professionals and specialists often move forward with an oral-only approach [24]. They move forward despite a lack of strong evidence that this approach is successful. Only when it becomes obvious that this approach has failed is sign language considered [16].

The approach of waiting to see positive results has been used with dyslexic children, and just as inadequately [25–27]. The difficulty of detecting dyslexia before a child has reached a certain level in grade school is given as the main rationale for why detection does not occur early, when it would be most effective [28, 29]. But recent developments in MRI studies in infants have changed possibilities for recognition of need and, hence, early support [30, 31].

The situation for deaf children, however, contrasts sharply with that of dyslexic children. The Centers for Disease Control’s *Early Hearing Detection and Intervention Guidance Manual* [32] recommends hearing screening of all babies no later than one month of age, and in most cases newborns are screened before leaving the hospital. Hence, families and the medical profession know that a child is deaf soon after birth. There is then no excuse for delaying supports with deaf children. A proactive approach of raising the child with sign language is both feasible and effective. Sign language is like a cognitive seat belt, protecting the child as a whole person [33]. Auditory deprivation in the early months of life, if not accompanied by sign language, is not just auditory deprivation but also deprivation of language – and language is essential for nourishing the human brain and partaking of humanity [34].

Given all this, why does the oral-only approach remain the dominant one? Delcenserie, Genesee, and Champoux [35] sum up the resistance to visual language acquisition for deaf children and the evidence against this resistance in this way:

Perhaps the most documented and empirical argument, the visual takeover hypothesis, suggests that exposure to sign language in deaf children may impair perceptual performance following cochlear implantation (e.g., Campbell et al., 2014). In other words, exposure to a visual mode of communication during the period of deafness would lead to auditory-to-visual plasticity, which can hinder the analysis of the auditory signal following implantation and lead to specific deficits in perceptual processes, including auditory frequency discrimination, auditory speech intelligibility, and speech recognition (Champoux et al., 2009; Giraud & Lee, 2007; Kral & Sharma, 2012; Turgeon et al., 2015; Zhou et al., 2018). However, recent findings by Mushtaq et al. (2020) show that there is a positive association between cross-modal plasticity and the speech perception outcomes of deaf cochlear implant users. In addition, increasingly, research suggests a lack of robust evidence to conclude that exposure to a visual language directly causes poor spoken language outcomes (Fitzpatrick et al., 2016) or negatively influences cross-modal plasticity (Lyness et al., 2013). Overall, the case for the visual takeover hypothesis does not seem sufficient to deprive deaf children of early exposure to sign language (see Pontecorvo et al., 2023, for more discussion of this issue).

We agree: the visual takeover hypothesis – that is, the claim that introducing a visual language will impair the implanted child’s acquisition of speech – has deterred the medical profession from advising families to introduce a sign language to their children.

Decades of research on the normalizing effects of sign language acquisition from an early age, however, refute the visual takeover hypothesis. Indeed, many studies have shown that signing does not hinder acquisition of a spoken language [36–38]; rather, the visual input of sign language supports all language learning and should be encouraged rather than discouraged [39]. Yet there remains adherence to the visual takeover hypothesis due to inadequate and outdated training about language, cognitive development, and the life-long impact of early language deprivation [40].

To remedy the situation, parents need better advice and better supports [41], and the medical profession globally needs to declare that all deaf children should receive a sign language as a health matter [42]. In this way, we can replace the process of watching so many deaf children fall further behind in language, learning, and social development with the process of assuring that our deaf children succeed.

The present narratives among professionals are so varying regarding the role of sign language in a deaf child’s language and cognitive development that they contribute to parents’ confusion and frustration. But parents are capable of more than they are given credit for when it comes to seeing what is happening with their child’s development trajectory; they are resourceful in finding ways to communicate with their children in spite of being told not to sign [43]. Many go ahead and learn a sign language so they can provide sign language input that is so beneficial to developing cognitive abilities and can create a rich socially nurturing world for their child [44–49]. Through sheer desire to do the right thing for their children, many educate themselves about sign language and use it at home [45], seeing the advantages even when the professionals do not always see them. They often come to the independent conclusion that shared reading activities with their deaf children in a sign language are an excellent way to promote language learning for the whole family and especially for their deaf children [48], a finding that research underscores [50–55]. In sum, as the wisdom of deaf people with lived experience confirms [56], parents of deaf children need an approach that does not wait to see if failure will ensue in an environment of insufficient linguistic input.

## Harms to the individual: linguistic deprivation

Newer research since that 2012 publication confirms and emphasizes that the failure to recommend that a deaf newborn be given access to sign language as soon as deafness is detected puts the child at risk of linguistic deprivation [19], which is implicated in wide-spread effects on brain development, and which can be permanent and, importantly, are preventable [57, 58]. Studies of children with hearing aids who are not taught to sign are far fewer than studies of children with CI who are not taught to sign – and there are so many variables at play, where different studies consider different variables, that comparing studies of the two groups is so complex as to presently be infeasible, if not invalid [59]. What we can confirm is that effects on brain development are evident in many deaf children with and without cochlear implants who are not taught to sign, and these effects correlate positively with defective or delayed development of memory organization [60], working memory [61], executive functions [62, 63], sequential processing [64], concept formation [65], numeracy abilities [66], statistical learning [67], and other neurocognitive skills [68]. Additionally, deaf children who are not taught to sign experience a range of difficulties when learning to read and write [69].

Linguistic deprivation remains an epidemic among deaf people. Though many succeed despite such deprivation, many more do not and end up presenting atypical or dysfluent sign language [70]. Linguistic deprivation and its effects continue to correlate with poverty, unemployment, or underemployment (where this claim is deduced from statistics on overall deaf poverty and employment, given the assumption that these figures are not based solely on prejudice regarding disability; see [71, 72]), involvement in criminal activity as victim or perpetrator [73], and poor life-long health outcomes [74]. The psycho-social harms of linguistic deprivation are devastating, but even delayed language access has serious harms [75–77]. This list leaves no doubt that sign language is a health need for deaf children [78, 79], and for deaf people in general (see the position paper of the World Federation of the Deaf [80]).

## Harms to the individual: CI surgery

There have been newer studies of CI surgery since our 2012 publication; here we report on a large and recent one by Parent and colleagues [81]. While we do not dispute its findings, we take issue with its conclusion.

Parent and colleagues’ study, of 5728 patients implanted in France between January 2012 and December 2016, found that CI surgery had a complication rate of 6.85%, which is far lower than the rate found in many other studies – a difference that the researchers go at lengths to account for. Further, the complication rate revealed little difference between adults and children, although complications rated “major” were significantly more common in the pediatric population than in the adult population. Some complications were decidedly easy to assess: the risk of device failure is higher in the pediatric population (1.16% versus 0.03%), where traumatic failures occur mostly in children [82, 83].

Parent and colleagues’ study does not report how judgments of certain other postoperative complications were assessed among pediatric patients. For example, the study reports that pediatric complications were mostly infections (18%) and device breakdowns (17%), while the major complications reported in adults were dizziness and scarring. Given that the complication rates in pediatric patients overall were 7.32% for children implanted before the age of 1 and 7.52% for children implanted between the ages of 1 and 2, we wonder how it was assessed whether children that young felt dizzy and were even aware of whether or not they had scarring. The authors themselves note that the symptoms of cochleo-vestibular complications are “difficult to detect” among children (and see [84]), suggesting a high probability of undercounted pediatric complications.

Parent and colleagues conclude, “Cochlear implantation is a safe technique with a low incidence of complications. The absence of increased risk in patients at the extremes of the age spectrum justifies offering this solution to all, without age limitation” [81].

This conclusion makes a blanket recommendation of CI without considering the age of implanted subjects. We consider that the evidence is not strong enough for this conclusion. We have asked elsewhere that regulatory agencies review guidance to address such conclusions. In the United States, the Federal Drug Administration (FDA) is the primary authority to regulate medical devices and the authority that the National Institute on Deafness and other Communication Disorders explicitly relies on [85], but it has shown insufficient oversight with regard to patient safety when approving many devices [86]. In particular, the FDA has not regulated CIs to the same extent as other medical devices [87]. In Europe the regulatory framework for medical devices was originally through the Confromité européenne (CE) marketing process and the Medical Device Directive 93/42/EEC [88, 89], where, again, regulation of CIs was problematic (e.g [90]). Since 2017, however, Regulation MDR (EU) 2017/745 [91] has taken over, and there have been a few improvements to the devices (such as resolving problems of magnet displacement; [92]), but postoperative complications still include an infection rate of 6.5% [93].

These complication rates are high by anyone’s standards. If we are going to ask deaf children to run these risks, we should at least ensure their access to sign language, as denying them this access would compound risks of language deprivation and ensuing cognitive deficits.

To be clear, we have no issues with CIs when the risks and concerns outlined in our previous papers and this paper are addressed. Unfortunately, we continue to observe that claims of success are reported in ways that at best obfuscate issues and at worst misrepresent results (see, for example, Hall and colleagues’ [21] critique of Geers and colleagues [94]). We continue to see few studies of the benefits of CIs when the child is in a signing environment, but existing ones show the combination of CIs with sign language is more advantageous than CIs alone (see [24, 95–97], as well as citations in the next subsection pertinent to the points made there). Thus CIs should not be used as a reason for delaying or withholding exposure to sign language, yet those involved in CI production and surgery continue a general silence about sign language benefits or advise parents outright that sign language will not benefit CI patients.

## Advantages of early sign language acquisition/learning

Additional research since our 2012 article has confirmed that the positive influences of signing from birth are pervasive and have widespread effects on brain development. In fact, deaf children who are early signers do not exhibit the cognitive harms discussed thus far with respect to memory organization [98], working memory [99], executive function [100, 101], sequential processing [98], language-based analogical reasoning [102], numeracy abilities [103], mathematical conceptualization [104–107], and statistical learning [108].

As for academic achievement, deaf children who have early access to sign language have been shown to use their knowledge of that language in learning to read the text of the ambient spoken language [109–111]. Signing skills are, likewise, among the strongest predictors of writing skills [112–116], even when the parents are hearing [117].

Parent signing at home with the child regardless of CI use is another significant factor in the educational success of deaf children [118, 119], particularly since it facilitates academic support. With the emphasis in many schools on inclusion in the classroom, an important contribution to the success of deaf children in that situation is the use of sign language interpreters for access, again regardless of CI use [120]. Thus, learning a sign language in the home before school is important because by the time the child is of school age, the ability to learn through an interpreter is critical. We cannot emphasize enough that parent-child bonding is most strongly established when sign communication in the home is used alongside the parents’ spoken language [121].

Overall, studies show that signing deaf children consistently outperform deaf children without sign exposure in academic settings [117, 122], and signing at home both aids academic performance and family bonding. As an additional benefit, outside the home and school, out in the social world, signing allows a deaf child to enter rich two-way communication encounters, which form the basis for making friends, exchanging information, making jokes, etc. – this is an enormous psycho-social benefit [123].

## Conclusions: needed change

New evidence confirms the findings of Humphries and colleagues [1], yet, unfortunately, we see little evidence of change in practice since then. Instead, the present policy of wait and watch as deaf children fail persists. This policy is reminiscent of two recent studies in HRJ and a forthcoming book, which point out untenable practices and social injustice: “Come back when you’re infected: pharmacy access to sterile syringes in an Arizona Secret Shopper Study, 2023” [124]; “‘They make it too hard and too many hoops to jump’: system of organizational barriers to drug treatment during epidemic rates of opioid overdose” [125]; *Designed to Fail: Why Racial Equity in School Funding Is So Hard to Achieve* [126]. As Snodden [127] outlines (in a study involving Canada – where the situation is very much like in the United States and many other places) the pattern of systemic and professional negligence and non-recognition of deaf children’s sign language rights reveals an absence of linguistic and social justice for deaf children.

We need to act, and the actions we need to take are clear. We must create approaches that move us towards supportive practices for deaf children and their parents, approaches that build on the wisdom of those most closely involved, including deaf people with lived experience and their parents. Detailed description of such approaches are found in Humphries and colleagues [41] (and see [75] for recommendations on mentoring families; see also [128] for recommendations on mentoring deaf scholars). Here we list major factors that must be included in such approaches.

- Updated and enriched knowledge regarding research on language and cognitive development and the harms of language deprivation in the professional development and training of those who advise parents of deaf children.
- Immediate and early support for parents in decision making about their deaf child and paths forward that include and emphasize the need for visual language input from detection of deafness onward.
- Immediate support for parents and their deaf child in learning a sign language at home and continued mentoring of deaf children throughout their education.
- Continuous shared narrative across the relevant disciplines (medicine, health, education, social services) that reinforce the fact that early sign language exposure is essential to preventing the risk of insufficient linguistic input for language and cognitive development.
- Protocols in all professions and disciplines that are inclusive of deaf people themselves in establishing ethical principles of practice that reflect and protect deaf people for the entire life cycle.
- Diversifying all relevant professions with the addition of deaf people in these professions.

These six changes are commitments necessary for designing an approach that succeeds. Implementing these changes, and quickly, will help prevent harm to the health and wellbeing of deaf children and will advance us all toward a more just society.

## Data Availability

No datasets were generated or analysed during the current study.
